# Exploring Use-Rates of and Scientific Evidence on Sutureless Devices in Aortic Valve Replacement: A Bibliographic Meta-Analysis and Clinical Considerations

**DOI:** 10.3390/jcm14124049

**Published:** 2025-06-07

**Authors:** Cristiano Spadaccio, Arnaldo Dimagli, Clayton J. Agler, Dane C. Paneitz, Stanley B. Wolfe, Antonio Nenna, Asishana A. Osho, David Rose

**Affiliations:** 1 Department of Cardiac Surgery, University of Cincinnati College of Medicine, 231 Albert Sabin Way, MSB 2474, Cincinnati, OH 45267, USA; aglercj@mail.uc.edu; 2 Department of Surgery, Columbia University, New York, NY 10027, USA; 3 Department of Surgery, Johns Hopkins University School of Medicine, Baltimore, MD 21287, USA; danepaneitz@gmail.com; 4 Department of Cardiothoracic Surgery, West Virginia University, Morgantown, WV 26506, USA; 5 Department of Cardiac Surgery, Ospedale Maggiore Della Carita, 28100 Novara, Italy; antonio.nnn@hotmail.it; 6 Department of Cardiothoracic Surgery, Harvard Medical School, Massachusetts General Hospital, Boston, MA 02114, USA; asishana.osho@mgh.harvard.edu; 7 Department of Cardiothoracic Surgery, Blackpool Teaching Hospital, Blackpool FY3 8NP, UK; mr.rose@nhs.net

**Keywords:** sutureless valve, aortic valve replacement, transcatheter valve replacement, minimally invasive cardiac surgery

## Abstract

Sutureless aortic valve replacement (SuAVR) has emerged as a potential alternative to conventional surgical aortic valve replacement (SAVR), particularly in minimally invasive settings. However, its global adoption remains limited, with a notable concentration of use and scientific production in select European countries. This bibliographic meta-analysis systematically reviewed 538 studies to assess the evidence landscape surrounding SuAVR, highlighting a predominance of observational data, sparse randomized controlled trials (1.3%), and significant geographical imbalances in research output. Europe accounted for 80% of publications, while North America contributed less than 10%. Key structural factors—including reimbursement policies, earlier regulatory approvals, and population characteristics—appear to influence SuAVR adoption. Despite procedural advantages such as reduced cross-clamp times, concerns over cost, pacemaker implantation rates, and uncertain long-term durability persist. Importantly, SuAVR may offer its greatest clinical value by facilitating minimally invasive surgery, a niche still underutilized worldwide. The limited randomized data and industrial focus on transcatheter approaches have further hindered widespread acceptance. Our findings underscore the need for high-quality comparative trials and standardized guidelines to define the role of SuAVR in modern valve therapy.

## 1. Introduction

Sutureless valves are devices designed to be surgically implanted without the need for anchoring sutures and have recently been considered a promising alternative to standard surgical valve replacement for aortic valve disease in selected cases [1]. The perceived main advantages of sutureless valve technology pertain to its ease of use, especially in minimally invasive settings, and to the reduction in ischemic surgical time compared to conventional sutured valves [2]. Hence, sutureless valves might be considered a valid adjunct in the surgical armamentarium in high-risk cases requiring prolonged or multiple combined procedures [1].

Nevertheless, there is no clear consensus within the surgical community regarding the actual role of sutureless devices in routine clinical practice, and there is a general degree of skepticism around its adoption [3,4,5]. Despite its complexity and the influence of numerous measurable and unmeasurable confounders, we aimed to explore and analyze the potential reasons behind the limited adoption of this technology, which has recently been regarded as a potential benchmark comparator for transcatheter aortic valve replacement (TAVR) in contemporary reports [6,7].

## 2. The Published Evidence

The literature on the outcomes of sutureless aortic valve replacement (SuAVR) seems to mainly derive from retrospective observational studies, with only two randomized clinical studies performed up to now. Hence, the “strength” of the evidence on SuAVR might not be compelling enough to convince the community of its adoption. To this scope, we performed a bibliographic meta-analysis of the available literature regarding SuAVR with the intent to dissect the characteristics and academic trends of the publications on this topic.

### 2.1. Methodology

A health science librarian performed a comprehensive literature search to identify English-language studies that reported on sutureless aortic valve replacement. An electronic search was performed using the Cochrane central register of controlled trials, Embase, Medline, Scopus, and Web of Science core collection using a combination of medical subject headings (MeSH/Emtree terms) and keywords for the concepts of sutureless valve and rapid-deployment valve published from inception through July 2023. Continuous variables were all non-normally distributed based on the Shapiro–Wilk test and were reported as medians and interquartile range (IQRs) and compared using the Kruskal–Wallis or Mann–Whitney U test. Categorical variables were reported as counts and percentages and compared using a binomial test. A *p* value of 0.05 was considered statistically significant. The analyses were conducted using R Statistical Software (version 4.2.1; R Foundation for Statistical Computing, Vienna, Austria).

We conducted a bibliographic systematic review and meta-analysis according to the Preferred Reporting Items for Systematic Reviews and Meta-Analyses (PRISMA) statement [8]. The objective was to critically assess the current evidence on sutureless aortic valve replacement (SuAVR), including clinical outcomes, surgical adoption patterns, and comparisons with standard surgical and transcatheter approaches.

The search strategy combined both Medical Subject Headings (MeSH) and Emtree terms, as well as relevant keywords, using Boolean operators for concepts related to “sutureless aortic valve,” “rapid deployment valve,” “aortic valve replacement,” “outcomes,” “minimally invasive,” and “TAVR.” Reference lists of selected articles and relevant reviews were manually screened to identify additional studies of interest.

#### Data Extraction and Analysis

Two independent reviewers screened the titles and abstracts for eligibility and performed a full-text review according to predefined inclusion criteria. Studies were included if they reported clinical or procedural outcomes of SuAVR (e.g., mortality, stroke, paravalvular leak, pacemaker implantation, cross-clamp time), compared SuAVR to standard SAVR or TAVR, or investigated specific contexts such as minimally invasive surgery, small annuli, or redo procedures. Disagreements were resolved by consensus.

The extracted variables included:

Study characteristics (year, design, sample size, country/region);
Affiliation type (academic, public, private);
Disclosure of conflict of interest;
Patient demographics and risk profile;
Surgical approach (conventional vs. minimally invasive);
Valve type and size;
Procedural metrics (cross-clamp time, CPB time);
Clinical outcomes (early mortality, complications, valve performance).

Where applicable, continuous variables were assessed for normality using the Shapiro–Wilk test. Non-normally distributed data were reported as medians with interquartile ranges (IQRs) and compared using the Kruskal–Wallis or Mann–Whitney U test, as appropriate. Categorical variables were expressed as absolute counts and percentages and compared using binomial tests. A two-sided *p*-value < 0.05 was considered statistically significant.

All statistical analyses were conducted using R Statistical Software (version 4.2.1; R Foundation for Statistical Computing, Vienna, Austria). Forest plots and heterogeneity metrics were used where pooling of data from comparative studies was feasible. Descriptive and comparative synthesis of key findings across different clinical contexts (e.g., MICS, redo AVR, small annulus) was performed to inform patterns of use and potential indications for SuAVR.

### 2.2. Results and Interpretation

**Figure 1:**
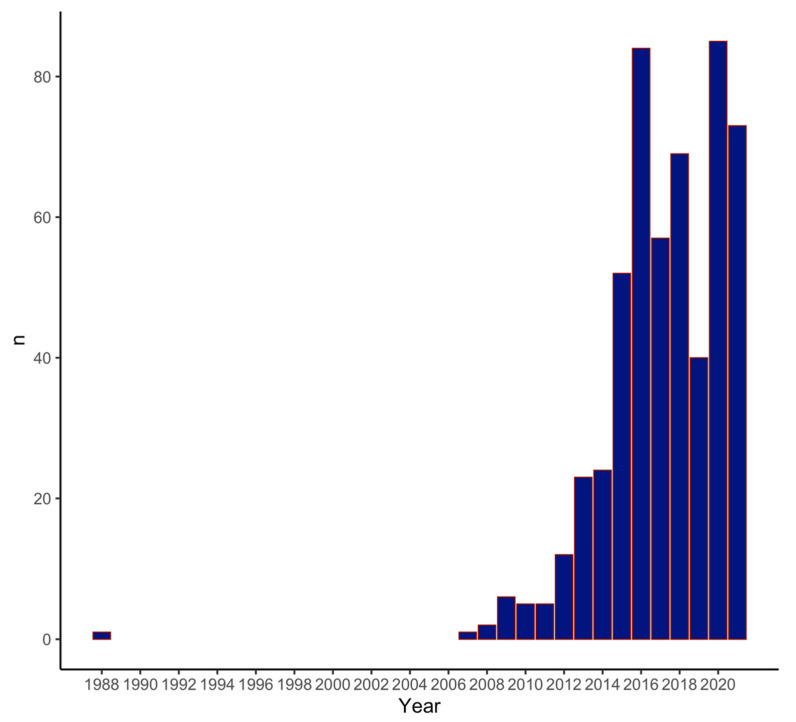
Annual publication trends in studies on sutureless aortic valve replacement (SuAVR) from 2007 to 2023. Peaks in 2016 and 2020 reflect growing interest, particularly in observational studies and European contributions. This trend mirrors increased device familiarity and early regulatory approval in Europe.

A total of 538 studies were included in this meta-analysis (observational and descriptive studies: 69%; randomized clinical trials: 1.3%; opinion pieces/editorials: 20%; reviews: 6.9%). Of these, 402 (75%) studies were published by academic centers, and 134 (25%) were published by non-academic centers. Interestingly, 76% of industry-sponsored studies were performed at academic centers (Table 1). As SuAVR is a relatively new concept, publications on the topic peaked in 2016 and 2020, and publications have steadily increased since 2007 (Figure 1).

When looking in greater detail at the geographic origin of the included studies, a marked imbalance favoring certain European countries could be observed. Italy emerged as the leading contributor, accounting for 147 studies (27.3%), followed by Germany (*n* = 113, 21.0%) and France (*n* = 36, 6.7%) (Table 2). Together, these three countries represented more than half (55%) of all publications on SuAVR. Additional European contributors included the United Kingdom (3.9%), Spain (3.5%), and a collective group of other European countries (17.3%), underscoring the dominant European presence in this research field (80% of the totality of SuAVR papers) (Table 2).

In contrast, contributions from North America were more limited. The United States accounted for only 9.9% (*n* = 53) of studies, and Canada for 2.0% (*n* = 11). Asian countries were even less represented, with Japan (2.6%), South Korea (1.7%), and other Asian nations (1.7%) contributing modestly. Turkey also contributed 11 studies (2.0%). Only two multicenter studies (0.4%) spanned multiple regions. Notably, there is a complete lack of representation from African, South American, and Oceanic institutions, indicating significant global gaps in both the adoption of and the academic discourse surrounding SuAVR.

There is not only a geographical delocalization of scientific production but also the type of studies differs across countries. While North American papers mainly consist of editorial or opinion pieces (26, 41%), European centers mainly publish observational studies (213, 50%). The majority of Asian studies on SuAVR consist of review pieces (13, 30%) (Table 1).

Funding disclosures were inconsistent. Over half of the studies (53%) declared no funding, while 38% did not report any funding status at all. Only 5.4% of the studies declared industry sponsorship, mostly in Europe. Foundation, society, or intramural grants were exceedingly rare (*p* = 0.368).

Regarding conflict of interest (COI) disclosures, 26% of studies reported a conflict, most of which were from Europe (28%). In contrast, only 4.7% of Asian studies disclosed conflicts of interest (*p* = 0.003). Not surprisingly, industry-sponsored studies are more likely to report a conflict of interest than non-industry-sponsored studies. However, there is a surprisingly high number of studies (176, 33%) where conflict of interest status is unknown (Table 1). This may suggest that a quota of the papers is published in journals with less strict submission criteria that do not require authors to disclose conflicts of interest. Additionally, lack of disclosure or details on COI could introduce a significant unmeasurable confounder in the perception and interpretation of the results. This is an issue pertaining not only to the SuAVR literature but to all the fields of scientific production.

The evidence in support of SuAVR is therefore primarily observational, with the majority of the literature relative to the experience of a single center or a single expert, particularly when minimally invasive surgery is taken into consideration. The dominance of observational and descriptive studies (69%) and the near absence of randomized clinical trials (1.3%) underscore a major limitation in the available literature. Evidently, the significant publication bias in observational studies undermines the scientific relevance of the findings, which could also be influenced by non-measurable confounding factors, such as the surgeons’ experience or predilection. Consequently, meta-analytic work available on the topic also reflects this significant heterogeneity [9], impacting the translatability of the results into real-life scenarios and potentially contributing to discouraging a larger-scale adoption of these devices.

In summary, this bibliographic meta-analysis identified a difference in the use-rate of SuAVR in Europe and the US, with the majority of original investigations and clinical outcome reports skewed towards European centers. On the other hand, the American literature mainly relates to single-arm trials to assess safety and Food and Drug Administration Investigational Device Exemption trials [10] and editorial pieces. Most of the literature review papers have been published in Europe and Asia. Such a geographical delocalization of the use of these valves and of the relative scientific production on this topic may mirror the non-univocal surgical consensus on SuAVR. Additionally, the imbalance between observational and randomized evidence may reinforce the ongoing clinical skepticism and limited adoption among some surgeons.

Variables include institutional affiliation, study type, focus, funding disclosure, and conflict of interest reporting, compared across Europe, North America, Asia, and multicenter studies.

Italy and Germany account for nearly half of all publications. This regional concentration underscores the European predominance in SuAVR research output.

A number of non-scientific structural drivers of this Europe-centered SuAVR landscape could be hypothesized. In fact, beyond differences in clinical evidence, several macro-level industrial and policy factors amplify the pronounced European predominance in SuAVR use and research. First, reimbursement frameworks may diverge and influence institutional decision-making. In most large European health systems—e.g., Germany’s Diagnosis Related Group (DRG) K60 for isolated aortic valve replacement—device-specific add-on payments mean hospitals recoup much of the higher valve cost, whereas in the United States, SuAVR is grouped under MS-DRG 266/267, together with conventional SAVR, providing no extra payment and leaving hospitals with a negative margin that discourages implantation [11]. Second, regulatory timelines appear to have favored earlier device familiarity in Europe. The Perceval and Intuity platforms obtained CE-mark approval in January 2011 and October 2011, respectively, but did not receive FDA pre-market approval until 11 January 2016 [12], giving a significant five-year advantage for European surgeons to build familiarity with the device, expertise, relationships with industry, center-of-excellence networks, and early academic–industry registries across Italy and Germany. Third, the value of SuAVR mainly resides in enabling minimally invasive surgery for the aortic valve. The feasibility of Minimally Invasive Cardiac Surgery(MICS) is often influenced by patient anatomy, chest conformation, body habitus, and comorbidities, with the ideal candidates being non-obese and relatively healthy patients. North America has an incidence of adult obesity higher than 40%, while in Asia, its incidence remains below 6%; in Italy, it ranges at around 10% and in Germany at around 20% [13]. Differences in population characteristics may also play a role in the adoption of MICS approaches, limiting eligibility and as a consequence the use-rate of SuAVR. So, it is possible to speculate that a favorable reimbursement profile, earlier regulatory clearance, concentrated industry–academic networks, and a reduced incidence of morbid obesity preventing minimally invasive surgical access created an ecosystem in which SuAVR is economically and logistically more attractive in Europe than in North America or Asia, magnifying the observed geographic skew in clinical utilization and scientific output.

When translating our findings into the clinical realm, it is possible to conclude that while SuAVR offers procedural efficiencies—particularly in MICS—it remains underutilized outside Europe, in part due to cost, regulatory lags, and perceived limited evidence strength. Clinicians should consider SuAVR primarily in cases where it enables minimally invasive access, especially in anatomically favorable patients. Institutional expertise and financial viability remain key determinants of adoption. Efforts should be directed toward generating randomized evidence, improving cost-effectiveness analyses, and standardizing indications to support guideline-based integration of this technology.

## 3. The “Time Factor”

When translating the scientific literature into the clinical scenario, the lack of solid evidence demonstrating the superiority of SuAVR vs. conventional surgical AVR (SAVR) can dissuade surgeons from the adoption of a novel technology. When analyzing the results of the only two randomized clinical trials available, the PERSIST trial has produced non-inferiority data in terms of mortality for the first year after implant only [14], and a recent sub-analysis has shown a significant reduction in major cardiovascular events and stroke but a higher rate of pacemaker implantation when compared to SAVR [15]. The CADENCE-MIS trial investigating rapid-deployment valves implanted in a minimally invasive fashion versus SAVR via full sternotomy showed a significantly reduced cross-clamp time and better valvular hemodynamic function in the rapid-deployment group at the cost of higher incidence of paravalvular leak [16].

The advantage related to reduced cross-clamp time and ischemic time is very debated. Although sutureless valves reliably shorten aortic cross-clamp time, definitive evidence that this reduction improves morbidity or mortality is still lacking [3,4,17,18,19,20,21]. The CADENCE-MIS randomized trial found that, despite markedly shorter cross-clamp times with minimally invasive SuAVR using rapid-deployment valves, cumulative cardiopulmonary bypass duration and clinical outcomes did not differ from those of full-sternotomy SAVR [22]. A small, non-industry-sponsored RCT in older patients with small aortic roots implanted with a totally sutureless prosthesis also showed reduced operative times without a mortality benefit, but it was under-powered to detect differences in major clinical endpoints [23]. In contrast, several retrospective observational studies associate prolonged cross-clamp or bypass times with higher risks of postoperative complications—such as renal or respiratory failure, low-output syndrome, atrial fibrillation, increased transfusion requirements, and longer hospital stays—and suggest a trend toward increased mortality [24,25,26]. A meta-analysis of randomized and propensity-matched comparative studies tailored to investigate the impact of reduced operative times on clinical outcomes showed similar 1-year survival rates among SuAVR and SAVR. However, SuAVR was associated with reduced postoperative complications such as atrial fibrillation and blood product transfusions in the SuAVR group, at the cost of increased pacemaker implantation [27]. Whether the appetite for the use of SuAVR is more driven by the ease of the implant and by the surgeon’s perception of reduced operative time in high-risk patients rather than an effective impact on clinical outcomes is difficult to demonstrate at present and would require ad hoc studies. However, it may explain the general tendency to favor these devices in minimally invasive surgery. This is in accord with our bibliography analysis, which demonstrates a higher utilization rate and greater scientific output from centers performing high volumes of minimally invasive cases. Nevertheless, the fear of pacemaker implantation, and, more importantly, the higher costs of the devices still represent the main deterrents to the adoption of SuAVR [28]. These considerations regarding the “time factor” remain debatable, especially in light of the increasing integration of automated knotting systems from minimally invasive platforms into standard practice and the evaluation of cost–benefit balance.

## 4. Other Surgical and Non-Surgical Factors

It is important to note that SuAVR has been a relatively recent introduction into the surgical field (FDA approval 2016). Therefore, data on long-term structural valve degeneration are not available and the longevity of these prostheses remains uncertain, despite promising results at mid-term [29]. With the recent publication of the excellent long-term result of standard SAVR from the COMMENCE trial with the Resilia glutaraldehyde-free technology [30], the decision on adopting sutureless devices might need further supportive evidence. The results of the CAVALIER trial (NCT01368666) are eagerly awaited in this regard.

In the same vein, the feasibility of valve-in-valve TAVR after SuAVR is a not definitively answered question and pertains to the different design of the currently available devices (rapid deployment or sutureless prostheses). The literature reporting rescue or treatment of failed SuAVR with TAVR is sparse but consistently reports its feasibility despite multiple challenges [31]. In totally sutureless valves, as the Perceval valve, the potential for deformation of the nitinol stent, underexpansion, underfolding, or leaflet malcoaptation secondary to oversizing may result in additional mechanisms of failure (migration, paravalvular leak, regurgitation) different than the most commonly occurring structural valve degeneration with stenosis, as in rapid-deployment valves [32]. Therefore, not only does the decision to implant a sutureless valve play a role but the choice of which specific prosthesis to use is also a key factor. Currently, the literature lacks tailored studies addressing this aspect. However, the post-market MISSION registry—Assessing Clinical Outcomes Using the Edwards Intuity Elite Valve System in Isolated AVR Using Minimally Invasive Surgery in a European Multicenter [33]—may soon provide valuable insights into this question.

Another important consideration is that SuAVR is predominantly utilized in minimally invasive cardiac surgery (MICS), a practice that remains limited due to various factors, including a lack of local expertise, logistical challenges, procedural volumes, and the associated learning curve. Given the relatively small number of centers performing MICS, the low adoption rate of sutureless devices may reflect the limited distribution and diffusion of minimally invasive surgery itself. Conversely, due to the skepticism among many surgeons regarding the benefits of sutureless technology, these prostheses are unlikely to be routinely used in centers that do not specialize in minimally invasive approaches.

In fact, it is possible to speculate from this literature analysis that the true clinical value of SuAVR may reside not in marginal improvements in cross-clamp time or simplified implantation per se but rather in its unique ability to facilitate minimally invasive surgical approaches. In conventional open surgery, placing 12–15 sutures around the annulus is routine and not particularly time-consuming for experienced surgeons. As such, many remain unconvinced that saving a few minutes justifies the higher cost or the need to master a new learning curve. However, in MICS—where the working space is constrained by limited access through small thoracotomies or upper mini-sternotomies—the technical challenges of valve implantation become significantly more relevant. In this context, the sutureless or rapid-deployment valve may offer practical advantages. Our bibliometric analysis reported here supports this paradigm. We observed that the majority of SuAVR-related publications come from centers that are both academic and specialized in minimally invasive approaches, particularly in Europe, where MICS adoption is more widespread. The high geographic concentration of SuAVR-related studies in countries like Italy and Germany, historically pioneering of MICS, further support this idea. In this niche, SuAVR may indeed offer a significant advantage, streamlining the procedure, reducing operative time, and avoiding the ergonomic limitations of complex suturing through narrow operative fields.

Lastly, the current industrial interest relies on TAVR and there is less appetite for corporate investment in MICS because minimally invasive SAVR is a much less reproducible and less scalable “product” when compared to TAVR. In fact, there is still no robust evidence that minimally invasive cardiac surgery offers superior outcomes over standard surgery, and the expertise in minimally invasive surgery is very variable, with a clear volume–outcome relation. Only a limited number of centers and surgeons truly master MICS, and its steep learning curve impacts both the reproducibility of results and the feasibility of treating large patient volumes. On the other hand, all the evidence on TAVR derives from robust randomized data, which are more able to influence and inform the guidelines. It is plausible to think that from an industrial perspective, TAVR represents a more attractive option, and this has led major heart valve manufacturers to redirect research efforts and investment towards transcatheter technologies and withdraw resources from the development of sutureless surgical platforms.

Current comparative studies between TAVR and SuAVR have demonstrated equivalent short-term mortality rates, with SuAVR showing a relatively better risk profile concerning paravalvular leaks and pacemaker implantation [1]. A number of different criteria play a role in the decision-making among the two approaches. Table S1 details the factors implied in patient selection and indications for TAVR or SuAVR (Table S1). However, it is crucial to recognize that TAVR and SuAVR are conceptually different procedures, and studies comparing their outcomes may be subject to significant biases. While randomized and real-world studies are necessary to clarify these differences, it is notable that 25% of surgeons would be reluctant to participate in randomized trials comparing TAVR with SuAVR, further complicating efforts to generate high-quality comparative data [28].

### Limitations

This study has several limitations. First, the bibliographic meta-analysis is based predominantly on observational studies, which are inherently subject to selection and publication bias. Second, the heterogeneity of study designs and endpoints precludes robust comparative analysis. Additionally, the inconsistent disclosure of funding sources and conflicts of interest raises concerns regarding transparency and potential bias in the existing literature. Finally, the inclusion criteria limited to English-language publications may have excluded relevant studies from non-English-speaking regions.

## 5. Conclusions

In conclusion, many factors can explain the relatively low adoption of SuAVR in the current era [1]. There is a clear geographical concentration of both the use of these devices and the related scientific literature, with the vast majority of studies originating from a limited number of European countries, particularly Italy and Germany. This uneven global distribution may weaken the generalizability and “strength” of the current evidence base. The perceived advantages in terms of cross-clamp time and ease of use are counterbalanced by the increased risk of pacemaker implantation and higher costs. However, these devices maintain a significant advantages in the minimally invasive cardiac surgery practice. Lastly, the lack of impulse from the industry in furthering the development of sutureless platforms in favor of transcatheter technologies has surely contributed to the current landscape.

## Figures and Tables

**Table 1:** Geographic differences in SuAVR publications across 538 studies.

Characteristic	Overall (*n* = 538)	Europe (*n* = 429)	North America (*n* = 64)	Asia (*n* = 43)	Multicenter (*n* = 2)	*p* Value
Affiliation						0.048
Academic	402 (75%)	310 (72%)	53 (83%)	37 (86%)	2 (100%)	
Non-Academic	134 (25%)	118 (28%)	10 (16%)	6 (14%)	0 (0%)	
Unknown	2 (0.4%)	1 (0.2%)	1 (1.6%)	0 (0%)	0 (0%)	
Study Type						<0.001
Observational	238 (44%)	213 (50%)	12 (19%)	11 (26%)	2 (100%)	
Descriptive	133 (25%)	98 (23%)	20 (31%)	15 (35%)	0 (0%)	
Opinion/Editorial	105 (20%)	76 (18%)	26 (41%)	3 (7.0%)	0 (0%)	
Review	37 (6.9%)	23 (5.4%)	1 (1.6%)	13 (30%)	0 (0%)	
Randomized clinical trial	7 (1.3%)	6 (1.4%)	1 (1.6%)	0 (0%)	0 (0%)	
Other	14 (2.6%)	10 (2.3%)	3 (4.7%)	1 (2.3%)	0 (0%)	
Focus						0.010
SuAVR data	443 (82%)	356 (83%)	51 (80%)	35 (81%)	1 (50%)	
SuAVR complications	81 (15%)	65 (15%)	12 (19%)	4 (9.3%)	0 (0%)	
Minor SuAVR focus	12 (2.2%)	7 (1.6%)	1 (1.6%)	3 (7.0%)	1 (50%)	
Other	2 (0.4%)	1 (0.2%)	0 (0%)	1 (2.3%)	0 (0%)	
Funding						0.368
Not Funded	287 (53%)	224 (52%)	39 (61%)	24 (56%)	0 (0%)	
Industry	29 (5.4%)	27 (6.3%)	2 (3.1%)	0 (0%)	0 (0%)	
Foundation/society grant	9 (1.7%)	7 (1.6%)	0 (0%)	2 (4.7%)	0 (0%)	
Intramural	4 (0.7%)	3 (0.7%)	0 (0%)	1 (2.3%)	0 (0%)	
Undeclared/unknown	207 (38%)	167 (39%)	23 (36%)	16 (37%)	2 (100%)	
Conflict of Interest						0.003
Yes	138 (26%)	121 (28%)	14 (22%)	2 (4.7%)	1 (50%)	
No	224 (42%)	169 (39%)	29 (45%)	26 (60%)	0 (0%)	
Not reported	176 (33%)	139 (32%)	21 (33%)	15 (35%)	1 (50%)	

**Table 2:** Country-wise distribution of SuAVR studies.

Country	Frequency	Percentage
Italy	147	27.30%
Germany	113	21.00%
France	36	6.70%
United Kingdom	21	3.90%
Spain	19	3.50%
Other European	93	17.30%
United States	53	9.90%
Canada	11	2.00%
Japan	14	2.6
South Korea	9	1.70%
Other Asian	9	1.70%
Turkey	11	2.00%
Multicenter	2	0.40%
Total	538	100%

## Data Availability

The original contributions presented in this study are included in the article/Supplementary Material. Further inquiries can be directed to the corresponding author.

## Supplementary Materials

The following supporting information can be downloaded at: https://www.mdpi.com/article/10.3390/jcm14124049/s1, Table S1: Clinical and procedural factors influencing the choice between SuAVR and TAVR. Considerations include anatomical suitability, comorbidities, institutional expertise, and procedural access route.

## Abbreviations

The following abbreviations are used in this manuscript:

CPB	Cardiopulmonary bypass
DRG	Diagnosis-Related Group
FDA	Food and Drug Administration
RCT	Randomized controlled trial
COI	Conflict of interest
SuAVR	Sutureless aortic valve replacement
TAVR	Transcatheter aortic valve replacement
SAVR	Surgical aortic valve replacement
MICS	Minimally invasive cardiac surgery
