# Supplementary material for: Exploring Use-Rates of and Scientific Evidence on Sutureless Devices in Aortic Valve Replacement: A Bibliographic Meta-Analysis and Clinical Considerations
Source: J Clin Med. 2025 Jun 7;14(12):4049. doi: 10.3390/jcm14124049 (PMC12194476; doi:10.3390/jcm14124049)
Supplement: Supplementary file 1 [file jcm-14-04049-s001.zip › jcm-3590242-supplementary.pdf]

Decision factor	When SuAVR is favored (over TAVR)	When TAVR is favored (over SuAVR)	Rationale/notes
**Age / life-expectancy**	65-80 y with ≥10 y expected survival	>80 y or <10 y life-expectancy	SuAVR offers surgical durability that matters only if lifespan is sufficient; TAVR minimizes procedural stress in the very old/frail.
**Operative risk (STS/EuroSCORE II)**	<4 % (low-intermediate)	>4 % (high) or prohibitive surgery	SuAVR still requires CPB and a mini-sternotomy/thoracotomy, unsuitable in prohibitive-risk patients.
**Anatomy & technical suitability**	• Unfavorable transfemoral access (severe ilio-femoral disease)<br>• Annulus size or morphology outside TAVR range<br>• High risk of coronary occlusion (small sinuses, low STJ)<br>• Heavy leaflet/annular calcification that jeopardizes TAVR sealing	• Favorable transfemoral access<br>• Adequate annulus dimensions within TAVR sizing matrix<br>• Low coronary-occlusion risk	SuAVR removes the native valve, avoids paravalvular leak from asymmetric calcium, and allows larger effective orifice area when annulus is small.
**Concomitant cardiac pathology**	Needs surgical correction (eg, CABG, ascending aorta, other valve) – SuAVR can be combined through minimally invasive approach	Isolated aortic stenosis with no additional lesions	In concomitant coronary pathology, staged TAVR and PCI could be considered in the case of prohibitive surgical risk.
**Prior chest interventions**	Not a contraindication but increased surgical risk	Presence of patent CABG grafts, prior chest radiation, mild-moderate chest deformity	Despite SuAVR enables chest access via anterolateral mini-thoracotomy or ministernotomy, presence of chest adhesions increases significant the risk, but in the context of redo surgery SuAVR can be preferrable to standard SAVR
**Frailty / severe systemic comorbidity (renal, hepatic, pulmonary)**	Absent or mild to moderate frailty	Marked frailty or end-organ dysfunction – favor catheter-based therapy	Frail patients may not tolerate even abbreviated CPB and general anesthesia required for SuAVR.
**Future coronary access needs**	Known or likely future PCI (eg, multivessel CAD) – SuAVR maintains unrestricted coronary ostia	Limited CAD, unlikely future PCI	High TAVR valve frames can impede coronary re-engagement, especially in small sinuses.
**Endocarditis**	Active or prior endocarditis – SuAVR allows radical debridement	Contraindication to TAVR	Removing infected tissue is essential; TAVR leaves native valve in situ.
**Patient-prosthesis mismatch concern (small annulus)**	SuAVR enables annular enlargement or supra-annular deployment with larger EOAs	Similar results when possible to accommodate appropriate TAVR size	SuAVR reduces mismatch risk without full root enlargement.

Table S1. Clinical and procedural factors influencing the choice between SuAVR and TAVR. Considerations include anatomical suitability, comorbidities, institutional expertise, and procedural access route.
